# Activation of AMPK/OPA1 pathway alleviates traumatic brain damage by regulating mitophagy

**DOI:** 10.1590/acb406925

**Published:** 2025-09-19

**Authors:** Hao Wei, Jiushan Liao, Wei Gao, Xiangzhong He

**Affiliations:** 1 Fuzhou First General Hospital Affiliated with Fujian Medical University – Department of Neurosurgery – Fujian – China.; 2 Luoyuan County Hospital of Fujian Province – Department of Neurosurgery – Fujian – China.

**Keywords:** Brain Injuries, AMP-Activated Protein Kinases, Optic Atrophy, Autosomal Dominant, Mitophagy

## Abstract

**Purpose:**

Mitophagy is an important process in brain damage, and the precise impact on a traumatic brain injury (TBI) model remains unclear. AMP-activated protein kinase (AMPK) regulates mitochondrial homeostasis and mitophagy, which are closely related to the remission of early brain injury. This study sought to explore the mechanism behind AMPK/optic atrophy 1 (OPA1) pathway in TBI via experimental verifications.

**Methods:**

TBI mouse model induced by weight-drop method was applied in this study. Neurological function tests, Nissl staining, TUNEL staining, and transmission electron microscopy were undertaken to assess the effects of mitophagy on the TBI model. Levels of apoptosis-related factors and mitophagy-related indicators were detected to further reveal the molecular regulatory mechanism of mitophagy in TBI.

**Results:**

Activation of mitophagy (MK-8722 or rapamycin treatment) reduced the severity of brain damage and mitigated neurological function deficits following TBI. MK-8722 treatment reduced neuronal apoptosis, improved neuronal mitophagy, effectively inhibited the expression of proteins Bcl-2 and Bax, and increased the expression of proteins Parkin, PINK1 and OPA1. Besides, MK-8722 improved TBI through accelerating the AMPK/OPA1 pathway, resulting in increase of mitophagy.

**Conclusion:**

This study is the first to pinpoint the AMPK/OPA1 pathway’s involvement in TBI and the mechanism of mitophagy, thereby providing a good foundation for future experimental studies.

## Visual Abstract

**Figure:**
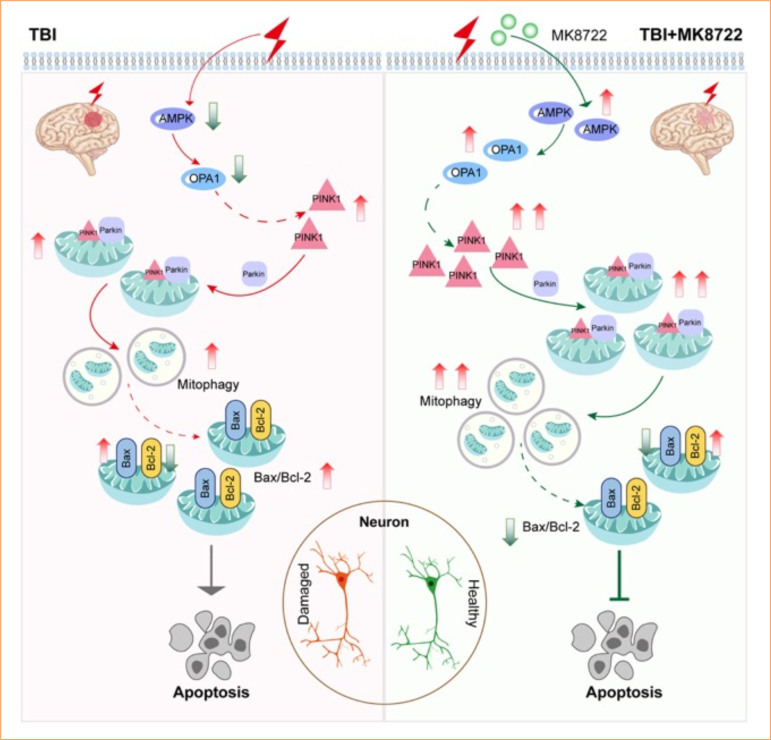
The activation of mitophagy by MK-8722 relieved neural damage by triggering the AMPK/OPA1 signaling pathway in neurons. Additionally, MK-8722 treatment enhanced neurological functions and brain electrophysiological activity following TBI.

## Introduction

Traumatic brain injury (TBI), resulting from external force impact, leads to structural or physiological dysfunction in the brain^1^. It ranks among the top causes of death and disability globally, placing substantial economic strain on patients^2^. Preclinical research has shown that anti-inflammatory agents have the potential to improve the prognosis of animal models of TBI, and clinical trial findings have been less encouraging^3^. Hence, identifying potential therapeutic targets for TBI is crucial due to its multifaceted pathology, including calcium overload, glutamate excitotoxicity, oxidative stress, neuroinflammation, and cell death^4^.

Maintaining proper mitochondrial dynamics is essential for neuron survival, as indicated by research showing that imbalances between fusion and fission processes occur in numerous brain injuries^5^. The clearance of dysfunctional mitochondria is pivotal for neuron viability. Autophagy selectively removes damaged mitochondria and preserved the equilibrium among organelle generation, protein synthesis, and cellular component degradation, which is crucial to TBI^6^. Earlier research has indicated that autophagy is pivotal in brain injury by eliminating dysfunctional mitochondria, which could otherwise trigger neuronal demise, thereby offering protection against secondary brain damage^7^. However, the specific mechanism of mitophagy action on brain damage remains unknown.

Optic atrophy 1 (OPA1) is embedded in the mitochondrial membrane, that is essential for regulating mitochondrial fusion. Reduced OPA1 levels are linked to the advancement of autoimmune myocarditis through the inhibition of mitochondrial fusion^8^. Knocking out OPA1 or inhibiting AMP-activated protein kinase (AMPK) pathway can block the promotion effect of melatonin on mitophagy^9^. OPA1 expression is medicated by AMPK signaling, and particularly, crosstalk between them is involved in the regulation of mitochondrial dynamics, such as mitophagy^10^. Neurons have high-energy demands, and their fate depends on mitochondrial function. AMPK activation is triggered by ATP depletion or glucose starvation.

AMPK, a widely preserved protein, plays a crucial role in promoting cell survival and organismal longevity by regulating energy balance and autophagy^11^. Therefore, modulating mitochondrial dynamics through AMPK activation could serve as a beneficial therapeutic approach in brain injury. However, the function of the AMPK/OPA1 pathway in brain injury needs further investigation.

Hence, this study aimed to explore the contribution of mitophagy to the progression of TBI. In addition, we explored the molecular mechanism of AMPK-OPA1 signaling in TBI, providing new ideas for the TBI therapy.

## Methods

### Animals

Adult BALB/c mice (male, 6–8 weeks old) were sourced from SPF (Beijing) Biotechnology Co., Ltd. The mice were provided ad libitum access to food/water. Meanwhile, animals were treated with a temperature range of 22°C ± 2°C and humidity fluctuating between 50 and 65%, in a 12-hour light-dark cycle. The Ethical Committee for Animal Welfare at Fujian Medical University approved these activities (Approval No.: IACUC FJMU 2024-0118).

### Animal grouping and traumatic brain injury model establishment

Twenty mice were randomly assigned to five groups, which included:

- Sham operation group (sham);
- TBI mice (model);
- AMPK agonist MK8772 treatment of TBI mice (MK8772);
- Mitophagy agonist rapamycin treatment of TBI mice (rapamycin);
- Mitophagy inhibitor 3-MA and MK8772 treatment of TBI mice (MK8772+3-MA).

Following one week of acclimatization to the diet, the mice underwent an 8-hour period of fasting. The TBI model was established by weight-drop method^12^. Briefly, the mice were anesthetized with 10 g/L pentobarbital sodium (i.p., 50 mg/kg) in prone position. After disinfecting the skin, a midline sagittal incision was performed on the skull to expose the right parietal bone. Next, a craniotomy (5-mm diameter) was conducted by an automated grinding drill, targeting the area between the bregma, 1.5 mm behind the coronal suture, and lambda, 2.5-mm lateral to the midline. The dura mater was left intact over the cortex, while a 20-g weight-drop device was released from a height of 20 cm to induce focal trauma on the cortex (the impact force causing injury is 200 g·cm). After that, a solution containing four or five drops of gentamicin sulfate was injected into the incision. Following this, the bone window was closed using bone wax, and the scalp incisions were stitched. The sham group of mice did not receive trauma treatment, and the rest of procedures remained consistent.

### Intervention treatment

Fifteen minutes before brain injury, MK8772 (i.p., 30 mg/kg) or rapamycin (i.p., 15 mg/kg) were given to the mice in MK8772 and rapamycin groups, respectively. The mice in the mitophagy inhibitor 3-MA and MK8772 group were treated with both MK8772 (i.p., 30 mg/kg) and 3-MA (i.p., 15 mg/kg). Mice in both the sham group and the model group received an equivalent volume of normal saline.

### Neurologic function testing

The neurologic functions of mice were measured using the modified neurological severity scores (mNSS) test on three days after trauma according to a previous study^13^. The neural function of mice was tested by sensory, motor, physiological reflex and balance capability, with a total score of 18. A higher score indicates more severe neurological function damage, and the completely normal score was 0.

### Preparation of brain tissue

The mice were flushed with a solution of phosphate-buffered saline (PBS) and 4% paraformaldehyde (PFA), followed by the removal of brain tissues after neurologic function testing. The brain tissue was immobilized in 4% PFA, then dehydrated with 30% sucrose until the bottom, and rapidly preserved at -80°C. Next, the brain tissues were embedded in paraffin blocks and sectioned into 6-μm slices. After dewaxing (100% xylene) and dehydration with gradient ethanol, the slices underwent microwave treatment in citrate buffer solution (cat# P0083, Beyotime, China) for 15 minutes to retrieve antigens. Subsequently, sections of the injured side of the cerebral cortex were excised, and the fragments were incubated in Dulbecco’s modified eagle medium (DMEM) medium containing 2 mg/mL papayase at 37°C for 30 min. The neurons were purified by immunoadhesion method. The cell suspension was added into the Petri dish coated with NCAM antibody, followed by being placed on a shaking for 1h, and the adhering cells were collected as neurons. The neurons were subsequently utilized for Nissl, TUNEL, and immunofluorescence staining.

### TUNEL *and Nissl staining*

Based on the manufacturer’s guidelines, apoptotic cells were detected through the TUNEL assay with the one step TUNEL apoptosis assay kit. Nissl staining involved treating sections with Nissl staining solution (cat# C0117, Beyotime, China) for 5 minutes, followed by rinsing with double-distilled water and mounting with Permount. Images were taken using a light microscope, and neuron counting was performed using ImageJ software, focusing on neurons exhibiting visible nuclei and relatively intact cellular morphology.

### Immunofluorescence staining

After antigen recovery, the brain tissues, after fixation, underwent treatment with 100% methanol for 10 minutes and were subsequently incubated in 1% bovine serum albumin for 1 hour. Following, brain sections were incubated with primary antibodies anti-Parkin and anti-translocase of outer mitochondrial membrane 20 (TOMM20) overnight at 4°C, followed by incubation with fluorescent-conjugated secondary antibodies for 1 hour at 37°C. Finally, 4’,6-diamidino-2-phenylindole (DAPI) staining was conducted, and the samples were examined using laser scanning confocal microscopy.

### Western blotting

Protein expression of AMPK, OPA1, PINK1, Bax, and Bcl-2 was measured via Western blotting. In brief, the obtained tissues were homogenized, and supernatant was obtained by centrifugation of homogenate. Proteins (25 μg/well) were quantified using a bicinchoninic acid assay, loaded onto 10% SDS-PAGE gels, transferred to polyvinylidene difluoride (PVDF) membranes, and blocked in 5% skim milk for 1 hour. They were then probed with primary antibodies against AMPK, OPA1, PINK1, Bax, and Bcl-2 for 1 hour at 37°C. Bands were visualized using an electrochemiluminescence reagent.

### Transmission electron microscopy

Cells were fixed overnight in 2.5% glutaraldehyde (dissolved with PBS), followed by post-fixation in 1% osmium tetroxide (pH 7.4) for 2 hours at 37°C. Dehydration was carried out using the gradient ethanol, and then infiltration with Spurr’s resin for tissue embedding. After polymerization at 60°C for 48 hours, samples were sectioned into 6-μm-thick slices using an LKB-I microtome, mounted on 200-mesh grids, and stained with uranyl acetate and lead citrate. Transmission electron microscopy (TEM) was conducted at 80 kV, with images captured directly from the microscope.

### Statistical analysis

Result data were reported as mean ± standard deviation, and differences between groups were compared using one-way analysis of variance (multiple groups) or Student’s t-test (two groups). A *p* < 0.05 indicated statistical significance. All statistical analyses were completed on GraphPad (version 7.0).

## Results

### MK8772 alleviated neurological impairment and improved neurological function deficits in mice following brain injury

**Figure 1:**
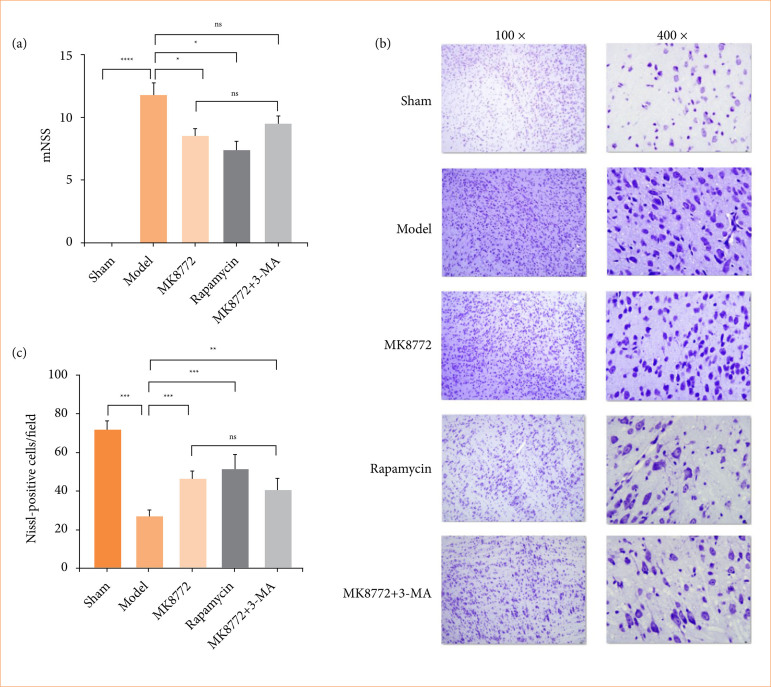
MK8772 relieved the extent of neurological impairment and alleviated motor function deficits in mice after brain injury. **(a)** Effect of different treatment on modified neurological severity scores. **(b)** Representative image of Nissl staining and statistical analysis of each mouse (100 × or 400 ×). Bars represent the mean ± standard deviation; one-way analysis of variance.

Three days after model establishment, mice exhibited significantly more severe neurobehavioral deficits compared to the sham group (*p* < 0.0001, Fig. 1a). Notably, MK8772 intervention led to a partial improvement in mNSS scores (*p* < 0.05, Fig. 1). Similarly, rapamycin also exhibited therapeutic benefit (*p* < 0.05, Fig. 1a). Inversely, combination treatment of MK8772 and 3-MA worsened the brain recovery with higher mNSS scores when compared with the MK8772 group. Similar findings were observed in Nissl staining (Fig. 1b). Quantitative results indicated that the number of Nissl-positive neurons in the model group was significantly reduced compared with the sham group. After administration of MK8722 and rapamycin, there was a clear reduction in neuronal loss compared to the model group. However, the protective effect of MK8722 on neurons was partially diminished after inhibition of mitophagy in TBI (Fig. 1c). These data suggested that the AMPK activator MK8722 may have a certain alleviating effect on neuronal injury after TBI in mice by regulating mitophagy.

*p < 0.05; ****p < 0.0001; ns: non-significant. Source: Elaborated by the authors.

### MK8772 treatment attenuated neuronal apoptotic death

**Figure 2:**
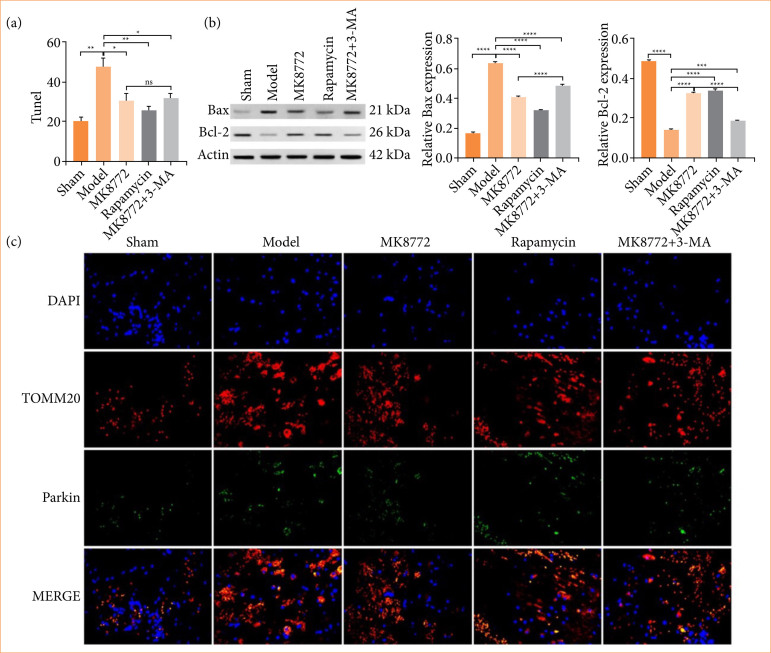
MK8772 treatment attenuated neuronal apoptotic death. **(a)** Representative quantification of TUNEL staining (n = 4 for each group). **(b)** Representative Western blot bands and quantification analysis of Bcl-2 and Bax. **(c)** Typical double immunofluorescence images of TOMM20 and Parkin.

TUNEL staining revealed a notable increase in TUNEL-positive neurons surrounding the trauma site in the model group, whereas MK8772 administration partially reversed this phenomenon (*p* < 0.05, Fig. 2a), and rapamycin treatment significantly inhibited cell apoptosis (*p* < 0.001, Fig. 2a). Compared with MK8772 treatment, cell apoptosis was slightly elevated in the MK8772 + 3-MA group. Furthermore, Western blotting revealed a significant elevation in Bcl-2 expression and a corresponding reduction in Bax expression following MK8772 treatment, compared to the model group (*p* < 0.001, Fig. 2b).

*p < 0.05; **p < 0.01; ***p < 0.001; ****p < 0.0001. Source: Elaborated by the authors.

### AMPK/OPA1 axis played a role in the neuroprotective effects of MK-8722-induced mitophagy

**Figure 3:**
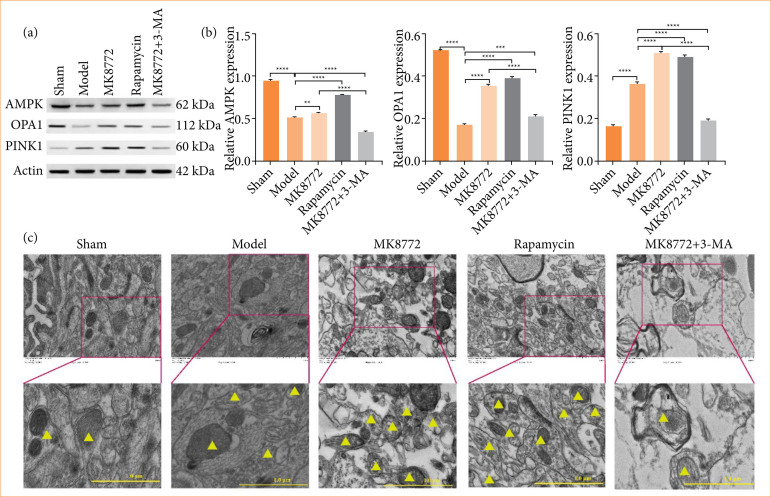
The AMPK/OPA1 axis participated in the neuroprotective effects of MK-8722-induced mitophagy. (a and b) Representative Western blot bands and quantification analysis of OPA1, AMPK, and PINK1. **(c)** Typical transmission electron microscopy images of morphological structure of autophagosomes and mitochondria in neurons (×10 magnification). Yellow triangle represents mitophagy.

AMPK: AMP-activated protein kinase; OPA1: optic atrophy 1; *p < 0.05; **p < 0.01; ***p < 0.001; ****p < 0.0001. Source: Elaborated by the authors.

Immunofluorescence staining exhibited that co-expression signals were reduced in TOMM20-labeled mitochondria (red fluorescence) and PINK labeled mitochondria (green fluorescence) after AMPK agonist MK8772 treatment. These results implied that MK8772 treatment can induce the abnormal expression of PINK1/Parkin pathway and affect mitophagy, but the combination treatment (MK8772 and 3-MA) led to an increase in comparison to the MK8772 group, as depicted in Fig. 2c. Western blot analysis indicated that following MK8772 treatment, Parkin1 expression rose compared to both the sham and model groups (*p* < 0.0001, as shown in Fig. 3a), revealing that MK-8722 could induce mitophagy. The TEM results were consistent.

Morphological structure of autophagosomes and mitochondria in neurons in the MK8772 group were unbroken when compared to the model group (Fig. 3c). Moreover, OPA1 and AMPK expressions significantly elevated in the MK8772 or rapamycin group, when compared to model group (*p* < 0.01, Fig. 3a). However, 3-MA addition significantly suppressed the expression of OPA1 and AMPK compared with MK8772 group (*p* < 0.0001, Fig. 3b). It demonstrated that AMPK/OPA1 axis may be contribute to the neuroprotective effects mediated by MK-8722-induced mitophagy.

## Discussion

TBI represents a diverse spectrum of disease manifestations characterized by significant neurological morbidity, and in severe cases, a high likelihood of mortality. Initial mechanical damage to neurons at the injury site results in significant neuronal loss, including glutamate excitotoxicity, disrupted ion balance, lipid peroxidation, nitric oxide release, and the release of inflammatory mediators^14^. Research has uncovered that in-vivo TBIs trigger the activation of PI3K/Akt/mTOR signaling pathways^15^ and regulate numerous physiological functions in the nervous system^16^. Additionally, the brain’s high-energy demands rely heavily on efficient glucose utilization and intact mitochondrial function. Sustaining mitochondrial activity and integrity are crucial for maintaining neural homeostasis^17^.

Furthermore, growing evidence suggests that autophagy remains elevated following experimental and clinical models of TBI, and activating the autophagy pathway plays a crucial role in conferring neuroprotective effects after TBI^18^. Post-treatment with Morin enhances memory impairment recovery by promoting autophagy following mild TBI^19^. Mitophagy could potentially mitigate neuronal injuries and dysfunctions by eliminating damaged mitochondria^20^. However, the pathology of TBI is complicated, and the function of mitophagy in TBI is unclear.

In this study, we explored the impacts of MK-8722 (an AMPK agonist) on TBI and underlying molecular mechanism responsible for these effects. Above findings indicate that the observed neuroprotective effect induced by MK-8722 was mediated via mitophagy, at least in part, through AMPK/OPA1 signaling in vivo. Our findings revealed that the activation of mitophagy by MK-8722 enhanced neurological functions, mitigated brain edema, and inhibited neuronal apoptosis at the traumatic sites following TBI. Moreover, the neuroprotective effect of MK-8722 is equivalent to the control reagent rapamycin (a mitophagy activator). Rapamycin also acts as a mTOR inhibitor, showing promise in alleviating TBI symptoms, including inflammatory reactions and epileptic responses^21^. Rapamycin boosts autophagic flux by inhibiting the PI3K/AKT/mTOR signaling pathway^22^.

In TBI, autophagy emerges as a protective mechanism, with rapamycin increasing beclin-1 protein levels to trigger autophagic responses. Furthermore, administering this mTOR inhibitor has been shown to enhance neurobehavioral functions, enhance neuronal survival, and mitigate inflammatory reactions following brain injury^23^. Therefore, we could infer that MK-8722 might act on the AMPK to regulate the mitophagy in TBI.

Increasing evidence emphasizes that secondary injury of TBI is closely related to apoptosis and autophagy^24^. It is well known that the brain has a strong demand for metabolic energy, of which neuron cells can consume about 75–80% of energy and are responsible for performing complex neurotransmission processes^25^. Importantly, the maintenance of normal functions of neurons mainly relies on the oxidative phosphorylation of mitochondria to produce ATP. Thus, mitophagy serves an important function in the central nervous system^26^. After TBI, damaged mitochondria release large amounts of pro-inflammatory factors and reactive oxygen species (ROS), causing severe oxidative stress and eventually cell death^27^. Meanwhile, these damaged mitochondria can be removed by activation of mitophagy, which helps maintain cellular homeostasis and respond to various brain injuries^28^. Hence, when the balance among oxidative stress, inflammatory response, and mitophagy is disrupted, the cell death process is initiated and accelerated accordingly, thus exacerbating the pathological reaction of TBI^29^.

Recent study has shown that PSMD14, as a therapeutic approach for improving TBI prognosis, can promote mitochondrial homeostasis by activating PINK1-mediated mitophagy, thus reducing ROS production and ultimately inhibiting neuronal apoptosis^30^. Consistent with this, we found that MK8772 can stimulate mitophagy by increasing PINK1 expression, thus elevating the Bcl2/Bax ratio to suppress neuronal apoptosis. The crosstalk between mitophagy and apoptosis is mediated by several key molecules, including BCL2 family members.

Previous study has further confirmed the causal relationship between apoptosis and mitophagy, suggesting that Parkin directly regulates the intrinsic apoptotic mechanism via inhibiting the activity of the mitochondrial apoptotic effector protein Bax^31^. However, our study lacks exploration of the mechanism underlying the participation of mitophagy in neuroprotective effects after TBI.

Intriguingly, PINK1 (a mitochondrial damage sensor) functions as a ubiquitin kinase, and its mediated phosphorylation of ubiquitin Ser65 residues and ubiquitin-like domains promotes the recruitment of latent Parkin on mitochondrial, and this association can eventually activate mitophagy^32^,^33^. Therefore, we speculated that MK8772 may attenuate TBI-induced neurological dysfunction by regulating ubiquitin-dependent mitophagy. This is an interesting aspect we will explore in the future.

Our study suggested that AMPK/OPA1 signaling pathway was involved in the neuroprotective effects of mitophagy, and that 3-MA (a mitophagy inhibitor) treatment could reverse the neuroprotective effect of the MK-8722. Moreover, 3-MA administration aggravated short-term brain damage after TBI. Besides, MK-8722 treatment further reduced the cell apoptosis at three days after TBI. Moreover, our results showed that the activation of the AMPK/OPA1 signaling pathway in neurons contributed to the neuroprotective effects of mitophagy.

OPA1 has been demonstrated to mitigate reperfusion injury in cardiac and cerebral tissues through promoting mitochondrial fusion. Chen et al.^9^ showed that melatonin improved mitochondrial fusion by promoting OPA1 expression, which ultimately reduced neuronal cell death. In the absence of OPA1, the protective effects of melatonin against cardiac reperfusion injury were rendered ineffective^34^. Our experiments revealed that MK-8722 boosted OPA1-associated mitophagy, resulting in decreased brain injury. Conversely, the administration of 3-MA diminished MK-8722’s impact on mitophagy and hindered its ability to activate mitophagy against brain injury.

Additionally, AMPK, a crucial energy sensor, takes part in the mitophagy that modulates cellular metabolism to uphold energy equilibrium^35^. Cui et al.^35^ found that melatonin reduced apoptosis in human umbilical vein endothelial cells by activating the AMPK pathway to stimulate mitochondrial fusion. In this study, MK-8722 upregulated the expression of AMPK protein, facilitated OPA1-mediated mitophagy, and enhanced mitochondrial clearance. Additionally, we employed a mitophagy inhibitor to assess MK-8722’s impact on mitophagy and observed that MK-8722 suppressed mitophagy via the AMPK/OPA1 pathway. Taken together, MK-8722 activated AMPK expression, leading to increased OPA1 levels and subsequent enhancement of mitophagy. This process ultimately decreased apoptosis and ameliorated neuronal damage. Our findings indicated that MK-8722 improved TBI-induced brain damage via AMPK/OPA1-mediated mitophagy.

Finally, our results indicated that activating AMPK could mitigate neural damage post-TBI, partly through the AMPK/OPA1 signaling pathway in neurons. Hence, stimulating AMPK/OPA1 holds promise as a therapeutic avenue for TBI management. Nonetheless, further in-vitro investigations are warranted to elucidate the precise molecular mechanisms involved. In addition, neuroinflammation is also a key pathological feature in TBI. Whether MK-8722 can slow down the release of pro-inflammatory factors is also a target that we need to pay attention to in the future.

## Conclusion

In summary, the activation of mitophagy by MK-8722 relieved neural damage by triggering the AMPK/OPA1 signaling pathway in neurons. Additionally, MK-8722 treatment enhanced neurological functions and brain electrophysiological activity following TBI. This suggested that mitophagy could serve as a promising therapeutic approach for TBI patients, with AMPK/OPA1 signaling representing a novel target for intervention.

## Data Availability

The datasets generated during and/or analyzed during the current study are available from the corresponding author on reasonable request.
